# One-time versus repeated abutment connection for platform-switched implant: A systematic review and meta-analysis

**DOI:** 10.1371/journal.pone.0186385

**Published:** 2017-10-19

**Authors:** Qing-qing Wang, Ruoxi Dai, Chris Ying Cao, Hui Fang, Min Han, Quan-Li Li

**Affiliations:** College &Hospital of Stomatology, Key Laboratory of Oral Diseases Research of Anhui Province, Anhui Medical University, Hefei, China; Virginia Commonwealth University, UNITED STATES

## Abstract

**Objective:**

This review aims to compare peri-implant tissue changes in terms of clinical and radiographic aspects of implant restoration protocol using one-time abutment to repeated abutment connection in platform switched implant.

**Method:**

A structured search strategy was applied to three electronic databases, namely, Pubmed, Embase and Web of Science. Eight eligible studies, including seven randomised controlled studies and one controlled clinical study, were identified in accordance with inclusion/exclusion criteria. Outcome measures included peri-implant bone changes (mm), peri-implant soft tissue changes (mm), probing depth (mm) and postsurgical complications.

**Result:**

Six studies were pooled for meta-analysis on bone tissue, three for soft tissue, two for probing depth and four for postsurgical complications. A total of 197 implants were placed in one-time abutment group, whereas 214 implants were included in repeated abutment group. The implant systems included Global implants, Ankylos, JDEvolution (JdentalCare), Straumann Bone level and Conelog-Screwline. One-time abutment group showed significantly better outcomes than repeated abutment group, as measured in the standardised differences in mean values (fixed- and random-effect model): vertical bone change (0.41, 3.23) in 6 months, (1.51, 14.81) in 12 months and (2.47, 2.47) in 3 years and soft tissue change (0.21, 0.23). No significant difference was observed in terms of probing depth and complications.

**Conclusion:**

Our meta-analysis revealed that implant restoration protocol using one-time abutment is superior to repeated abutment for platform switched implant because of less bone resorption and soft tissue shifts in former. However, future randomised clinical trials should be conducted to further confirm these findings because of the small samples and the limited quality of the original research.

## Introduction

The focus of dental implant therapy has shifted from functional therapy in the 1980s and prosthetic-driven therapy in the 1990s to biological-driven therapy since the 2000s. Biological-driven implant therapy does not only recover the function of soft and hard peri-implant tissues but also maintains their aesthetics and long-term stability[1].

Biologic width (BW) of dental implant is critical to the quality and stability of peri-implant structure[2]. BW around implant comprises sulcus epithelium, junctional epithelium, and connective tissue, and its physiological formation initiates crestal bone resorption and remodelling once the implant is exposed to the oral environment[3]. Crestal bone remodelling process is a biologic response to create space for new attachment of supracrestal fibres to the implants for biologic soft tissue seal[4]. BW formation and maturation mainly occurs between the sixth and eighth week of wound healing[5]. BW determines the minimum dimension from the junctional epithelium to attainment of connective tissue to ensure an ideal seal and to provide protection from mechanical and external biological agents. Any external agent invading the BW would induce a response from the epithelium that migrates beyond this agent, trying to isolate it[6].

The microgap and micromotion between the implant body and the abutment, position of the inflammatory response of the soft tissue seal to oral environment and the distribution of stress from loading through the implant are considered to be main mechanisms of such bone resorption[7–10]. The restorative protocol is one of the factors associated with the abovementioned mechanisms[11].

In standard clinical implant restoration protocol, healing abutment /provisional crown is connected to the implant body once implant is exposed to oral cavity. Before final prosthesis fabrication, provisional abutments must be disconnected and reconnected several times for impression making, metal framework try-in, delivery of definitive pre-fabricated standard or customized abutments, and final prosthesis. In the repeated abutment protocol, the dis/re-connected manipulation of abutment may mechanically injure the soft tissue barrier, and may introduce bacteria and other contaminants into the implant–mucosal barrier to induce inflammation. Therefore, dis/re-connected abutment manipulation may disturb the implant–mucosal barrier, that is, disturbance of the zone of ‘junctional epithelium and connective tissue integration’, and further affect the marginal peri-implant tissues, including the peri-implant bone, and finally affect the stability of the peri-implant tissue[12]. On the contrary, another restorative protocol is ‘one-time abutment’, which means definitive abutment is connected to the implant once implant is exposed into the oral environment. The definitive abutment is retained during all the procedures of the final prosthesis fabrication and no healing abutment is needed.

In literature, confusing is used to describe the process of implant restoration involving abutment dis/reconnection. It is often described as ‘repeated abutment’, ‘removable abutment’, ‘abutment dis/reconnections’ or ‘provisional/healing abutment’. In this review, this method is referred to as ‘repeated abutment’. By contrast, the process of implant restoration involving the use of definitive abutment to connect implant body without dis/reconnection manipulation is often referred to ‘one-time abutment’, ‘one-abutment at one time’, ‘one abutment-one time’, ‘one-time abutment placement’, ‘non-removal of an abutment’ and ‘definitive/final/standard abutment connected without removing during implant placement surgery or at stage two surgery’. In this review, ‘one-time abutment’ was used to denote the above confusing names.

Implant design is also one of the facts associated with the bone resorption. Implant with platform switching is widely used in clinics today. Platform switching is based on the use of abutments with smaller diameter compared with the platform diameter of the implant, thereby creating a mismatch between both components at the level of the implant–abutment interface[13, 14]. Thus, the microgap is more distant to the first bone-implant contact, and by shifting the implant–abutment interface medially, the deleterious impact of the implant–abutment microgap on the peri-implant bone can be reduced. This configuration results in a circular horizontal step, and the increase in the horizontal implant surface may move the connective tissue inflammatory infiltrate away from the bone crest and reduce the loading stress in the crestal portion of the bone[15]. The efficacy of this method to preserve crestal bone levels has scientific evidence[16–20].

Given that repeated abutment dis/reconnections may jeopardise the advantages of platform switching, the use of a definitive abutment connected to implant body without removal during the restoration process should be an additional strategy for using platform-switching implant system. However, conflicting findings regarding its superiority to repeated abutment have been reported in animal study and clinic study[21–32]. Whether the one-time abutment protocol has the advantages to maintain the stability of peri-implant tissue over the protocol of repeated abutment when platform-switched implants are used needs to be further confirmed.

Some case reports and randomised controlled clinical trials have been conducted since 2010[21–32]. Although a narrative review revealed that intentional abutment disconnections and reconnections can induce apical repositioning of the soft tissues and marginal bone resorption, it did not provide any statistical analysis after comparing such method with repeated abutment[33]. Therefore, this systematic review aimed to combine all the current clinical trials to compare clinical difference between one-time abutments and repeated abutments with a meta-analysis.

## Materials and methods

The population, intervention, comparison, and outcome of this systematic review are as following[34]:

**Population or participants:** Patients that need implant restorations

**Intervention:** One-time abutment during implant restorations

**Comparison:** Repeated abutment during implant restorations

**Outcome:** Peri-implant tissue changes (clinical and radiographic aspects)

### Inclusion and exclusion criteria

#### Inclusion criteria

1. Randomised controlled trials (RCTs) and clinical controlled trials (CCTs) with a minimum of six-month duration of follow-up after abutment connection to implant body
2. Studies that compared peri-implant tissue changes in subjects with one-time abutment and subjects with repeated abutment
3. Implant systems with the characteristics of internal connection and platform switching
4. Participants are ≥ 18 years of age and without chronic periodontitis and history of systemic disease

#### Exclusion criteria

1. Case report, conference proceedings, reviews, animal studies and *in vitro* study
2. Implant systems with the characteristics of external connection
3. Duplicate publication

### Search strategies

A comprehensive electronic search was conducted in the following electronic databases: Web of Science, Pubmed and Embase (updated until August 15, 2017). The following search terms were used in Pubmed and change depending on the rules of each database:

1. ((‘provisional’ AND Dental Abutment [Mesh]) OR (‘temporary’ AND Dental Abutment [Mesh]) OR (‘healing’ AND Dental Abutment [Mesh]) OR (‘repeated’ AND Dental Abutment [Mesh]) OR (‘disconnection’ AND ‘reconnection’ AND Dental Abutment [Mesh]) OR (‘removal’ AND Dental Abutment [Mesh]))
2. ((‘non-removal’ AND Dental Abutment [Mesh]) OR (‘final’ AND Dental Abutment [Mesh]) OR (‘definitive’ AND Dental Abutment [Mesh]) OR (‘standard’ AND Dental Abutment [Mesh]) OR (‘immediate’ AND Dental Abutment [Mesh]) OR (‘one-time’ AND Dental Abutment [Mesh]) OR (‘one time’ AND Dental Abutment [Mesh]))
3. #1 AND #2

### Selection of study

**Fig 1:**
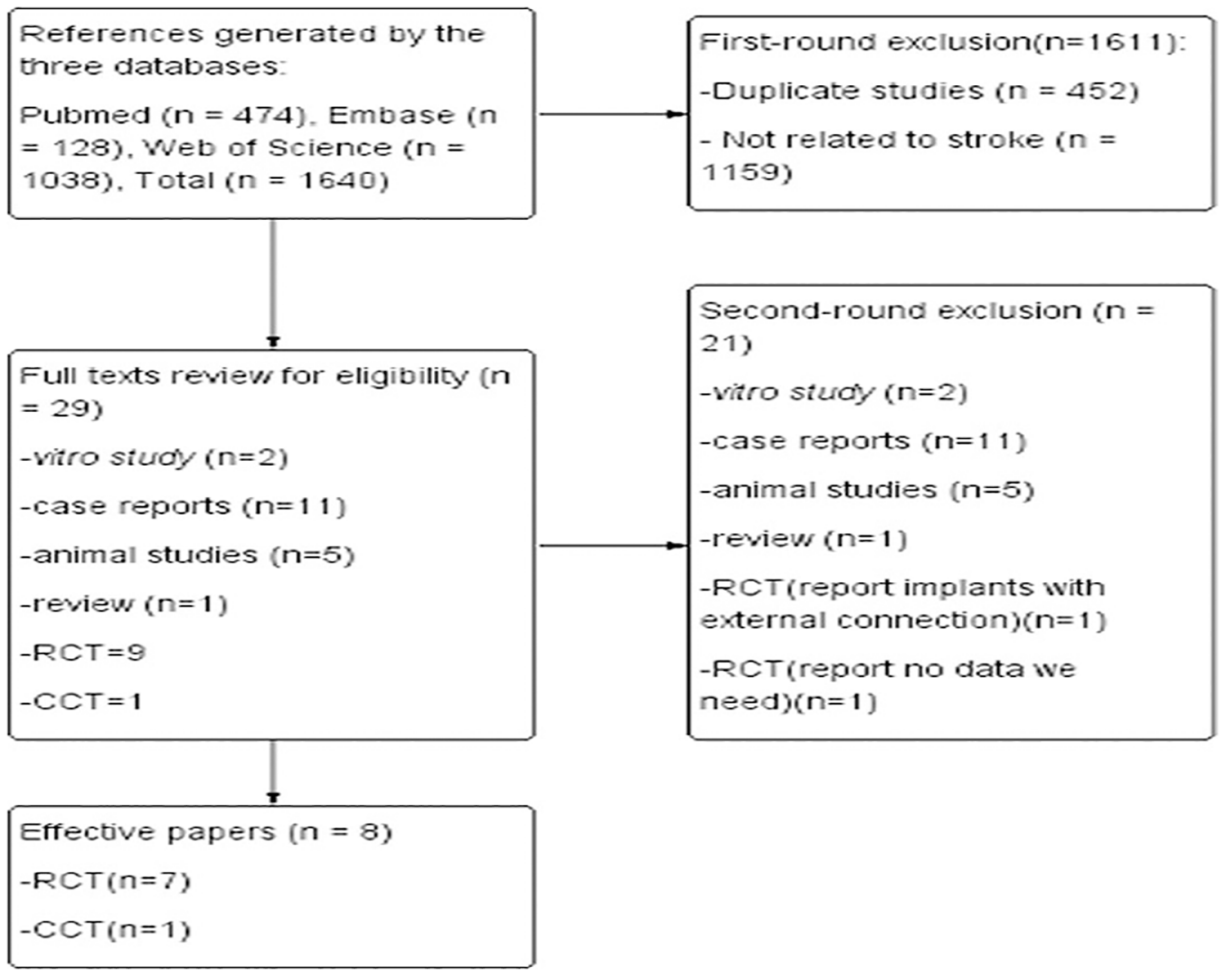
Flowchart of manuscript screened.

The titles and abstracts of all articles acquired from the electronic search were screened independently by two authors. Irrelevant studies were discarded. The full text of potentially relevant articles obtained from the above search strategies were further screened by two reviewers. Papers were excluded if they were case report, conference proceedings, reviews, animal studies and *in vitro* studies. Discrepancies were resolved by discussion between the reviewers. Only RCTs and CCTs that compared one-time abutment with repeated abutment and reported data on peri-implant tissue changes with a follow-up period of at least six months were selected and formed the base of this systematic review. The flow diagram was made using Review Manager Software according to the process of study selection (Fig 1).

### Data collection and meta-analysis

From the studies included in the final analysis, the following data were extracted (when available): year of publication, study design, implant surface, single- or multicentre study, number of implants, patient age, follow-up, antibiotic prophylaxis, use of mouth rinse, time from implant surgery to final restorations, implants sites, the relationship between the implant platform and the crest bone, stage of implant surgery, implant system, implant design, abutment manipulation, type of final prosthodontic retention, primary and secondary outcome, and main conclusions.

The outcomes in the meta-analyses are as follows:

#### Primary outcomes

*Vertical peri-implant bone changes*: Difference in vertical distance between implant platform level and the most coronal bone contacting with implant surface (mm). Vertical peri-implant bone level was recorded as average value in medial and distal site and measured using periapical radiographs with parallelization system and under standard calibration.

*Peri-implant soft tissue changes*: Difference in vertical distance between peri-implant highest buccal mucosa margin in maxillary or lowest buccal mucosa margin in mandible to the most coronal part of the prosthesis (healing abutment, the provisional restorations or the definitive restorations) (mm).

#### Secondary outcomes

*Probing depth*: Difference in probing depth (mm)

*Postsurgical complications*: Difference in occurrence of pain, swelling, mucositis after surgery, or sensory disturbance

Dichotomous data were expressed as risk ratios (RRs) with 95% confidence interval (CI). Continuous data were expressed as standardised mean difference (SMD) with 95% CI. Forest plots for each meta-analysis presented the original data of outcomes (displayed as blocks) with their respective CIs (displayed as lines), heterogeneity statistic (I^2^) and the pooled data of outcome (as rhomboid). Summary effects were calculated in both random- and fixed-effect models using Review Manager 5.3. The time points of bone changes and complications were from implant surgery, whereas the peri-implant soft tissue shifts and probing depth changes were from loading after mucosal detumescence. The plus sign represents bone growth or coronal soft tissue shifts, whereas the minus sign indicates bone resorption or apical soft tissue shifts. The outcomes of vertical bone changes were divided into subgroups according to their respective follow-up periods. Meta-analyses were performed when the included studies reported the same outcome measurements with the similar follow-up periods.

### Quality assessment

The qualities of the included RCTs and CCTs were assessed in accordance with the recommendations of Cochrane Collaboration. The following terms were used to determine biases in the included studies: 1) selection bias refers to sequence generation and allocation concealment; 2) performance and detection bias refer to blinding of participants and outcome assessors; 3) attrition bias refers to incomplete outcome data; 4) reporting bias refers to selective report of outcome.

## Results

The main outcomes were presented in tables and figures. Supporting information including consort checklist, PRISMA checklist and data availability checklist was presented from S1, S2 and S3 Tables.

The study selection process is summarised in Fig 1. The search generated a total of 1640 papers. After screening the titles and abstracts in the first round, 29 papers were identified as relevant. After retrieving the full text, 21 of them were further excluded. Five of them were animal studies[7, 35–38], two were in *vitro* studies[39, 40], one is a review[33] and 11 were case reports and case series[32, 41–50]. One study [29] reported implants with external connection and non-platform switching. One study [30] did not provide relevant data that we need. In this study, authors compared a friction fit abutment (test group) with a conventional healing abutment (control group), and in both groups, abutments were dis-/reconnected several times. A total of eight papers[21–28] were finally included in this systematic review.

### Description of the studies

The detailed information of the eight eligible clinical trials is listed in Table 1.

**Table 1:** Detailed information of included studies.

Studies	Canullo et al. 2010	Degidi et al. 2011	Degidi et al. 2014	Gandi et al. 2012	Grandi et al. 2014	Koutouzis et al. 2013	Luonge et al. 2015	Molina et al. 2016
Sex ratio (male/female)	16/19	equal	NM	11/17	9/16	7/9	33/47	NM
Patient age and range (year)	53.0	49.3	39.1	51.2	56.5	56.7	56.6	23.0–71.0
Smoking (cigarettes)	<10	<10	<10	<10	=<20	<10	NM	<10
Number of implants	25	48	53	56	25	21	128	55
Study design (RCT or CCT; single or multicenter)	RCT (multicenter)	CCT (NM)	RCT (NM)	RCT (multicenter)	RCT (multicenter)	RCT (NM)	RCT (multicenter)	RCT (NM)
The relationship between implant platform and bone crest	At the bone crest level	Beneath the bone crest	Beneath the bone crest	At the bone crest level	Beneath the bone crest	At the bone crest level	Beneath the bone crest	At the bone crest level
Implants sites	Premolar area of maxilla	Posterior mandible	Canine to canine maxillary	Partially dentate	maxilla or mandible from second premolar to second premolar	Posterior to the maxilla or mandible	Partially edentulous	Posterior to the maxilla or mandible
Stage of implant surgery	Immediate extraction sockets	Healed sites	Immediate extraction Sockets	Healed sites	Immediate extraction sockets	Healed sites	Immediate extraction sockets or healed sites	Healed Sites
Implant system	Global implant	Ankylos	Ankylos	JDEvolution JdentalCare	JDEvolution JdentalCare	Straumann Bone level	Ankylos	Conelog Screwline
Implant designs	Tapered implant with internal octagonal connection and platform-switching	Tapered implant with internal tapered connection and platform-switching	Tapered implant with internal tapered connection and platform switching	Tapered implants with internal tapered connection and platform- switching	Tapered implants with internal tapered connection and platform- switching	Tapered implants with internal tapered connection and platform-switching	Tapered implants with internal tapered connection and platform-switching	Tapered implants with internal conical connection and platform-switching
Implant surface	sand-blasted and acid-etched	grit-blasted and acid-etched	Grit-blasted, and acid-etched	NM	double acid-etched treated surface	NM	NM	NM
Abutment manipulation (control: times)	NM	3	3	3	3	3	3	3
Antibiotic prophylaxis	Amoxicillin and clavulanic acid	Beta-lactam for 5 days	Beta-lactam for 5 days	Beta-lactam for 6 days	Amoxicillin and clavulanic acid for 6 days	Amoxicillin for 6 days	Amoxicillin for 6 days	Prophylactic Therapy
Mouth rinse	0.12% chlorhexidine	NM	NM	chlorhexidine	0.2% chlorhexidine	0.12% chlorhexidine	0.2% chlorhexidine	0.12% chlorhexidine
Type of final prosthodontiucs retention	NM	Screw-retained	NM	Cement-retained	Screw-retained	Cement-retained	NM	Screw-retained
Period from surgery to final restoration	3 months	6 months	6 months	3 months	4 months	3 months	At least 3 months	8-10weeks
Follow-up (month)	36 months	36 months	24 months	12 months	12 months	6 months	7 months	12 months
Vertical bone loss (mm) (one-time/repeated)	-0.34/-0.55	NM	1.905/1.685	-0.091/-0.433	-0.108/-0.583	-0.13/-0.28	-0.08/-0.09	-0.603/-1.279
Horizontal bone changes (mm) (one-time/repeated)	NM	0.225/0.104	0.205/0.09	NM	NM	NM	NM	NM
Soft tissue shift (mm) (one-time/repeated)	NM	NM	-0.35/-0.59	NM	NM	0.12/0.18	NM	0.547/0.242
Change of probing depth (mm) (one-time/repeated)	0.02/0.03	NM	NM	NM	NM	NM	NM	0.893/0.488
Complications (events/total)	NM	one-time: 2/24 repeated: 2/24	one-time: 2/24 repeated: 2/29	NM	One-time: 1/12 repeated: 1/13	NM	One-time: 2/40 repeated: 3/40	NM
Main conclusions	Vertical bone resorption: repeated>one-time (*p*<0.05)	Vertical bone resorption: repeated>one-time (*p*>0.05) Horizontal bone changes: epeated<one-time (*p*<0.05)	Vertical bone resorption: repeated<one-time (*p*>0.05) Horizontal bone changes: repeated<one-time (*p*<0.05) Soft tissue recession: repeated>one-time (*p*<0.05)	Vertical bone resorption: repeated>one-time (*p*<0.05)	Vertical bone resorption: repeated>one-time (*p*<0.05)	Vertical bone resorption: repeated>one-time (*p*<0.05) Soft tissue remolding: repeated>one-time (*p*>0.05)	Vertical bone resorption: repeated>one-time (*p*>0.05)	Vertical bone resorption: repeated>one-time (*p*<0.05) Soft tissue remolding: repeated<one-time (*p*>0.05)
NM: not mention	NA: not acquired							

#### Study design and patient features

A total of 197 implants were placed in the one-time abutment group, whereas 214 implants were included in the repeated abutment group. Among eight eligible studies, seven studies were RCTs[21, 22, 24–28]and one study[23] was CCT. Four studies [21, 24, 25, 27] were multicentre RCTs, whereas the rest [22, 23, 26, 28]were unspecified its study sites. The minimum duration of follow-up period was 6-months. All of the eligible studies reported patient age, and most of the participants were middle-aged persons. All participants in the eligible studies were systemic healthy subjects without diabetes, osteoporosis and other systemic disease which may influence the quality of implantation. Six studies reported that participants smoked less than 10 cigarettes, one study less than 20 cigarettes, one study did not mentioned smoking.

#### Installation site and restoration characteristics

The implant systems included *Straumann Bone Level (Straumann*, *Switzerland)*; *Camlog*, *Conelog Screw-Line (Basel*, *Switzerland)*; *JDEvolution*, *JdentalCare (Modena*, *Italy)*; *Global Implants (Sweden & Martina*, *Padua*, *Italy)* and *Ankylos (Friadent*, *Germany*). All implants are with an internal implant–abutment connection and platform switching. Information regarding the length, diameter and shape of the implants were also provided. One study[22] evaluated implants inserted in the anterior maxillary regions, five evaluated implants placed in posterior regions[21, 23, 25, 26, 28], and two did not mentioned[24, 27]. Four studies reported implantation in healed sites[23, 24, 26, 28], whereas three studies reported immediate implantation[21, 22, 25], and one study reported implants placed in immediate extraction sockets or healed sites[27]. All of the implants underwent two to six months of healing before final restorations. Four studies reported that implants were placed beneath the bone crest [22, 23, 25, 27] and four at bone level [21, 24, 26, 28]. Three studies reported screw-retained restorations [23, 25, 28], two reported cemented [24, 26], three did not mention [21, 22, 27]. In all studies, abutments were disconnected and reconnected thrice, including impression making, the metal framework and biscuit fitting and the delivery of the definitive prosthesis.

### Quality assessment

**Fig 2:**
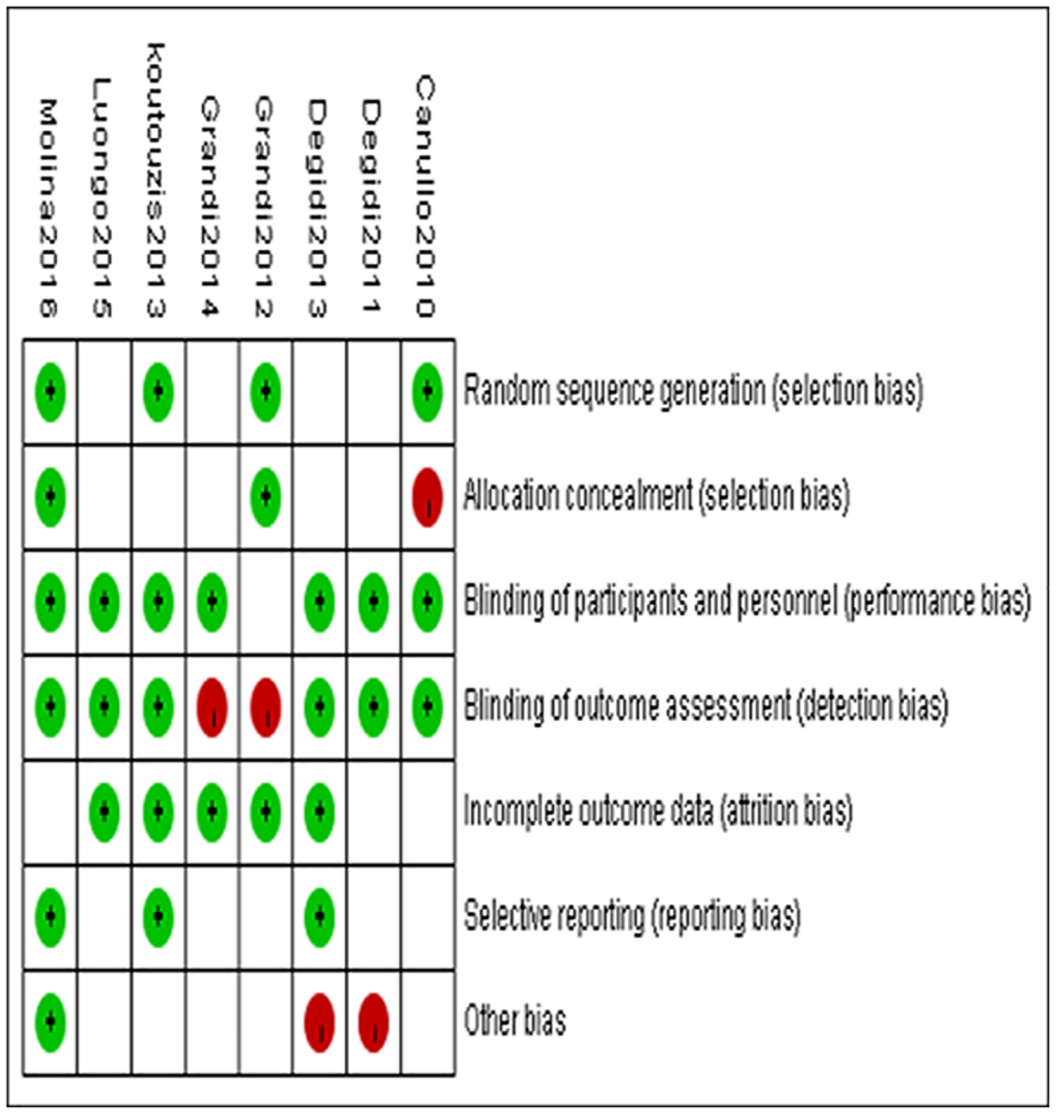
Risk of bias summary.

Each trial was assessed for risks of bias, and the results are summarised in Table 2, and Fig 2. Among the eight studies that met the inclusion criteria, four studies clearly described the random sequence generation[21, 24, 26, 28], and two studies described the allocation concealment clearly[24, 28]. Seven studies reported that participants were blinded [21–23, 25–28]. Six studies reported that outcome assessors were blinded [21–23, 26–28]. For the incomplete outcome, five studies described some exclusions of participants, in which they need not to be considered as leading to missing outcome data [22, 24–27]. Three studies [22, 26, 28] reported peri-implant soft and hard tissue changes. The drop-out rates were less than 45% in all studies. The common reasons for attrition were failure to achieve oral hygiene, lack of initial insertion torque and unsuitable extraction sockets. All of these conditions can result in bias.

**Table 2:** Quality assessment of the articles.

	Canull etal. 2010	Degidi et al. 2011	Degidi et al. 2014	Grandi et al. 2012	Grandi et al. 2014	Koutauziset al. 2013	Luongoet al. 2015	Molina et al. 2016
Sequence Generation	Low	Unclear	Unclear	Low	Unclear	Low	Unclear	Low
Allocation Concealment	High	Unclear	Unclear	Low	Unclear	Unclear	Unclear	Low
Blinding of participants and personnel	Low	Low	Low	Unclear	Low	Low	Low	Low
Blinding of outcome	Low	Low	Low	High	High	Low	Low	Low
Incomplete Outcome	Unclear	Unclear	Low	Low	Low	Low	Low	Unclear
Selective Reporting	Unclear	Unclear	Low	Unclear	Unclear	Low	Unclear	Low
Other bias	Unclear	High	High	Unclear	Unclear	Unclear	Unclear	Low

### Meta-analysis

Six out of eight papers selected in this review can be pooled for meta-analysis. One study reported vertical bone change that was measured from implant platform level to the crest of peri-implant bone, rather than to the most coronal bone contacting with implant surface[23]. Another study reported that bone level was measured at the mesial, the buccal, the distal, and the palatal sites using CBCT rather than periapical radiographs[22]. The bone level of every follow-up at the mesial, the buccal, the distal, and the palatal sites was presented separately in tables. Moreover, they did not report the mean values of bone level changes with standard deviation in tables. So those two studies were not included in the meat-analysis of bone change.

**Fig 3:**
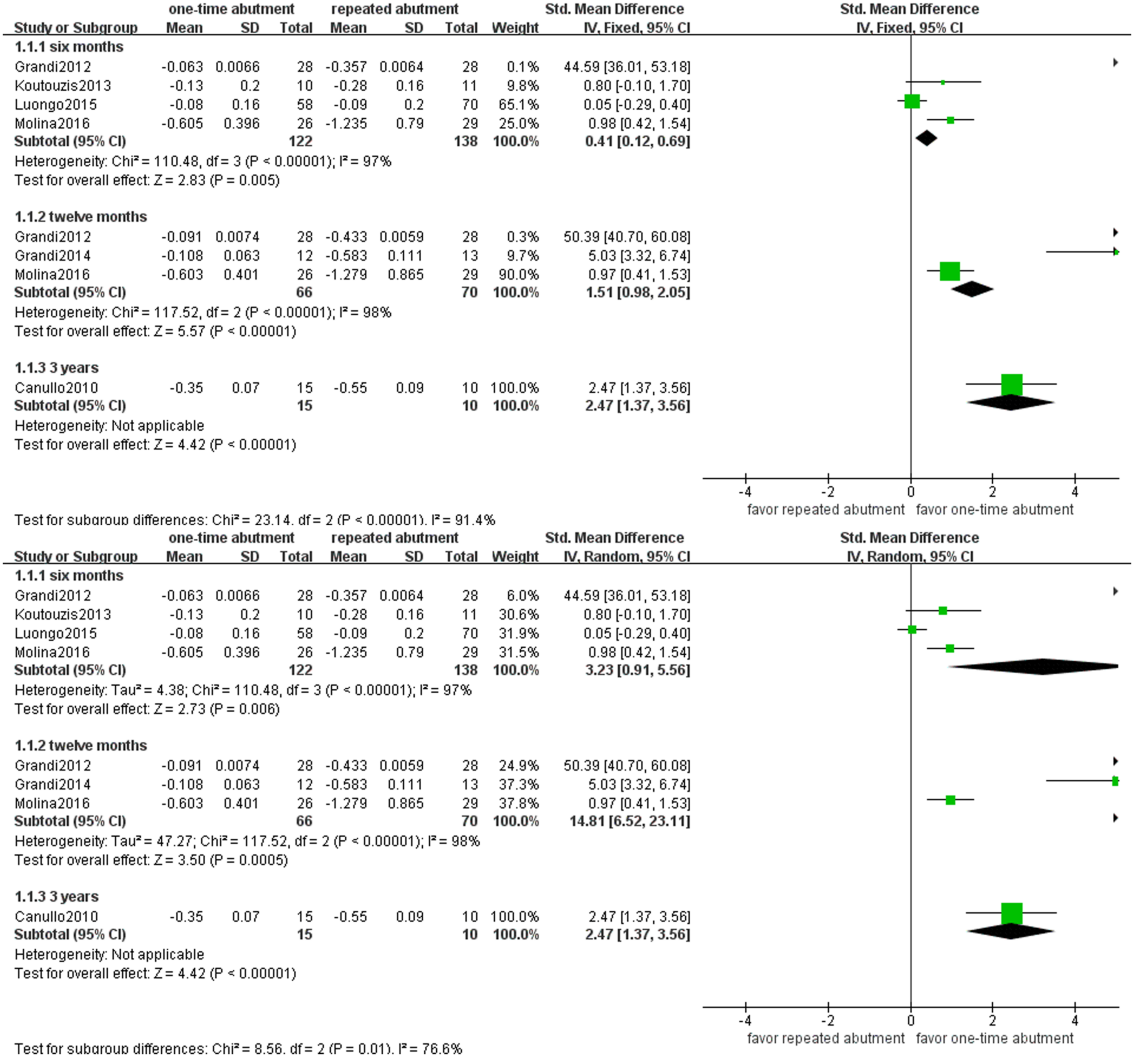
Forest plot of vertical bone changes.

**Fig 4:**
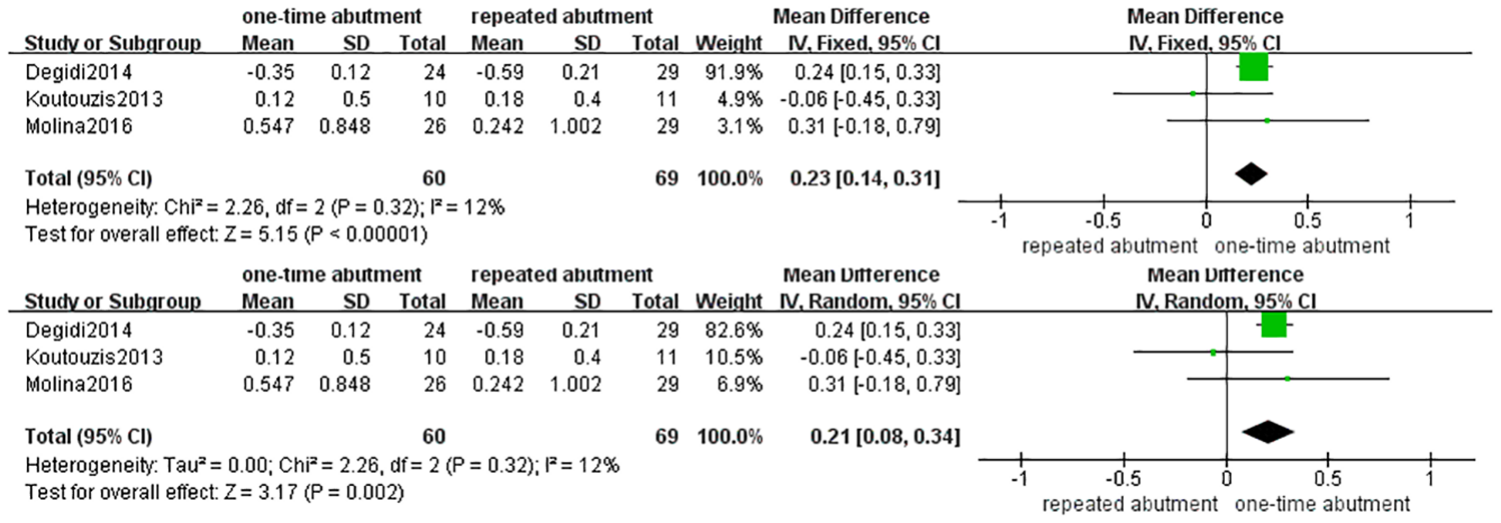
Forest plots of peri-implant soft tissue shifts.

**Fig 5:**
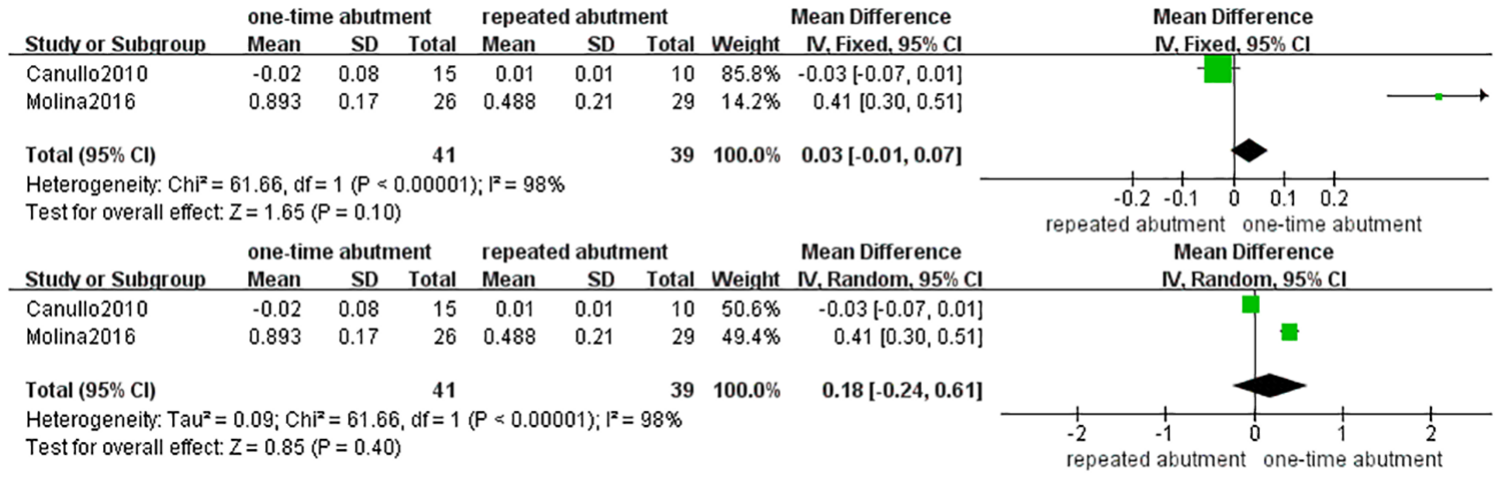
Forest plots of probing depth.

**Fig 6:**
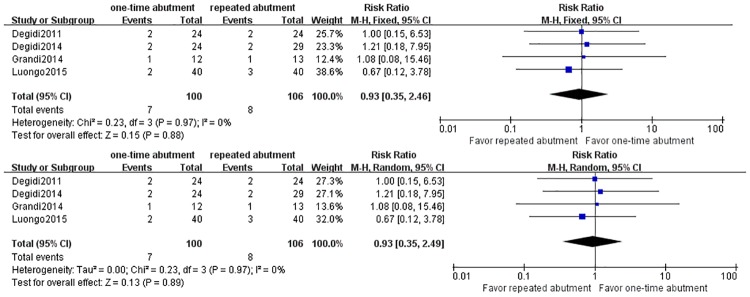
Forest plots of post-surgical complications.

The results of fixed- and random-effect models are presented in Figs 3–6. Studies on vertical peri-implant bone changes were divided into three subgroups- 6month, 12month and 3-year subgroups and presented in Fig 2. For the 6-month subgroup, four studies reported vertical bone change[24, 26–28]. Therefore, four studies that included a total of 260 implants were used for meta-analysis, and the SMD (95% CI) of vertical bone resorption was 0.41(0.12, 0.69) in fixed model (p<0.01) and 3.23 (0.91, 5.56) in random model (p<0.01). For the 12-month subgroup, three studies with a total of 136 implants reported vertical bone change[24, 25, 28]. Therefore, three studies were used for meta-analysis, and the SMD (95% CI) of vertical bone resorption was 1.51(0.98, 2.05) in fixed model (p<0.00001) and 14.81 (6.52, 23.11) in random model (p<0.001). For 3-year subgroup, one study reported vertical bone change[21]. Therefore, one study with 25 implants was used for meta-analysis, and the SMD (95% CI) of vertical bone resorption was 2.47 (1.37, 3.56) in fixed model (p<0.00001) and 2.47(1.37, 3.56) in random model (p<0.00001). The results of subgroup analysis indicated that one-time abutment can significantly reduce vertical bone resorption compared with repeated abutment.

The meta-analysis of peri-implant soft tissue shift was demonstrated in Fig 4. Three studies were included in the meta-analysis of peri-implant soft tissue shift[22, 26, 28]. The SMD of peri-implant soft tissue shift was 0.23(0.14, 0.31) in fixed model (p< 0.00001) and 0.21 (0.08, 0.34) in random model (p<0.01). The analysis revealed that one-time abutment significantly increases coronal peri-implant soft tissue shift compared with repeated abutment.

The meta-analysis of probing depth is illustrated in Fig 5. For the outcomes of probing depth, data with standard deviations of the mean differences between one-time and repeated abutment were provided by two studies[21, 28]. One study reported the results of 6 and 12months of follow-up time[28]. One study reported the findings of 18-month and 3-year outcomes after implant loading[21]. The data regarding the 12- and 18-month follow-up were combined in our meta-analysis because of the similar follow-up period. For probing depth, the SMD of increase in probing depth was 0.03(−0.01, 0.07) in fixed model (p>0.05) and 0.18(−0.24, 0.61) in random model (p>0.05).

The meta-analysis of postsurgical complications is demonstrated in Fig 6. The meta-analysis did not find any significant difference between one-time and repeated abutment for risk of postsurgical complications [RR: 0.93(0.35, 2.46), p>0.05 in fixed model and 0.93(0.35, 2.49), p>0.05 in random model].

## Discussion

An experimental study in a dog model demonstrated that the disconnection and reconnection of healing/provisional abutments can compromise the mucosal barrier and induce an apical migration of the connective attachment and remodelling of the underlying bone. Abutment manipulation resulted in a mechanical injury to the soft tissue barrier that had to re-establish more apically, causing a marginal bone resorption. However, the implant used was Brӓnemark system with an external implant–abutment connection and non-platform switching[35]. This implant system is not commonly used today compared with internal connection. An experimental study reported the effect of abutment disconnection on bone resorption by comparing platform-switched *vs* non-platform-switched implants with internal connection. The implants with a platform-switched design show less peri-implant bone resorption during the healing process than non-platform-switched implants[38]. However, the influence of abutment dis/re-connection on bone resorption with platform switching is controversial in animal model. A study reported that the shift from a healing abutment to a permanent abutment resulted in the establishment of a transmucosal attachment. The dimension and quality of transmucosal attachment in the repeated abutment protocol did not differ from those formed in one-time abutment protocol. In the study, a platform switching implant system was used in dog model, and the abutment shifting protocol was similar to that used in clinics[51]. Another study also reported that the connection/disconnection of platform switching abutments during prosthetic phase of implant treatment does not induce bone marginal absorption and only affects the connective tissue portion, which becomes shorter particularly in thin biotypes of implant BW. In the study, the abutments were connected/disconnected five times (at 6/8/10/12/14 weeks) after implant placement and evaluated at nine months after implant placement in dog model[12]. However, a study recently reported that repeated manipulation of abutment with platform switching may be associated with dimensional changes of peri-implant soft and hard tissues. The researchers connected abutment to implant at the time of implant placement surgery, and abutment dis/reconnection was repeated twice at four and six weeks after the surgery and observed at eight weeks in dog model[7]. Therefore, conflict results were found in animal study on “repeated abutment protocol” vs “one-abutment protocol”.

Our meta-analysis revealed that one-time abutment resulted in less vertical peri-implant bone resorption and soft tissue changes compared with repeated abutment. The microgap between implant and abutment interface is the main negative outcome for crestal bone resorption. The microgap between implant and abutment may lead to micromotion and bacterial leakage, resulting in tissue inflammatory infiltration. This phenomenon can result in increased peri-implant bone resorption and the disruption of peri-implant soft tissue attachments[35, 36]. In the classical repeated abutment protocol, once the implants are exposed into the oral environment, healing abutments will be fixed for approximately three weeks to three—four months prior to the insertion of standard or custom-made final abutments. Compared with prefabricated final abutment, healing abutment often provides less friction fit and performs less preloading force (often <10 N.cm) to implant body. By contrast, one-time abutment protocol, in which a prefabricated final abutment is connected to the implant body once exposed into the oral environment, performs more preloading (often 30 N.cm). Prefabricated final abutment demonstrated better matching interface between implant and abutment, resulting in less microgap and micromotion than repeated abutment protocol with a healing abutment. Thus, one-time abutment protocol may result in less bacterial leakage and inflammation. Moreover, one-time abutment avoids repeated dis/reconnection of healing/provisional abutments and reduces the disruption of soft tissue attachments. Previous studies have shown that repeated disconnection and reconnection of healing/provisional abutments can disrupt the established mucosal seal and lead to an apical shift of the connective tissue attachment and remodelling of the underlying bone[35]. The repeated manipulation to attach abutment to implant can also cause bacterial leakage, which further accelerates peri-implant tissue disruption. Therefore, one-time abutment may result in less vertical bone resorption and soft tissue changes.

For vertical bone resorption, our meta-analysis revealed that one-time abutment resulted in significantly less vertical bone and soft tissue changes, but the difference is of slight clinical significance. The reason for the slight difference may be that the tissue change around the implant is a very complex process, and the abutment manipulation is not the only factor to affect the change. Similarly, platform switched implant shows less bone resorption compared to non-platform switched implant, but the difference is also not obvious, and two type of implant system are both proved successful in clinics[16, 52]. In addition, limited study design and relative small sample size should also be considered. Moreover, it remains unclear whether the loss of bone would increase as the times of dis/reconnection of abutment increase. A recent RCT study was carried out to argue this issue. In their study, in the test group, prefabricated final abutment was connected to the implant body and tightened to 30Ncm when the implant body was exposed at the second-stage surgery. At each step of the prosthetic fitting, the abutment was removed and tightened to 30Ncm again. In the control group, conventional healing abutment was used when the implant body was exposed at the second-stage surgery, and the healing abutment was removed and reconnected at each step of the prosthetic fitting. The results showed that alveolar bone loss was significantly greater in control group than that in test group. Although the study design lacks another control group of one-time abutment protocol, it highlighted that the placing a stable and well frictional fit abutment is of more importance in reducing marginal bone remodelling than abutment manipulation[30]. Therefore, the interpretation of statistical significant difference between one-time and repeated abutment in clinical use must be cautious and the effect of dis/reconnection of abutment during the implant restoration needs to be further examined.

For probing depth, no significant difference between one-time and repeated abutment was observed. The reasons for similar probing depth may be due to absence of peri-implantitis occurring in follow-up time, and in all the included studies, the implant systems were similar with a platform-switching; thus, they may have similar BW. The BW remains stable for both one-time and repeated abutment protocols. Although our analysis revealed that one-time abutment resulted in less bone resorption than repeated abutment, the difference of resorption may be insignificant to cause significant difference in probing depth.

For postsurgical complications, no significant difference between one-time and repeated abutment was observed. The occurrence of pain, swelling and mucositis was associated with surgical procedures and postsurgical care rather than disconnection and reconnection of abutment. All the included studies reported similar implant surgery procedures and postsurgical care.

Significant heterogeneity was detected among individual studies in meta-analyses on peri-implant vertical bone resorption and soft tissue shifts. The presence of statistical heterogeneity may be due to the low power of statistical test because only few studies were included in the aforementioned meta-analyses. Therefore, selecting to use either fixed- or random-effect model should not be based on the tests of heterogeneity, and results from both fixed- and random-effect model analyses are presented. Of note, both fixed- and random-effect models were consistent in their statistical inferences.

Given the limited number of eligible studies, meta-regression analyses were not conducted to address some important confounding factors, including implant level, implant sites and prosthodontics retention type, associated with outcomes. Implant levels can be under crestal bone or at bone level. Some studies reported that implant levels at crestal bone may lead to marginal bone loss[53, 54], whereas some studies reported that implant levels may not jeopardise the position of the peri-implant tissue[55, 56]. Implants can be placed in healed sites with different duration after tooth extraction or fresh extraction sockets. Some studies reported that placing an implant immediately after tooth extraction can enhance hard and soft tissue maintenance[57, 58], whereas some studies reported that implants placed in fresh extraction sockets show more adverse effects than those inserted in mature bone[59, 60]. Implant restorations can be retained *via* cements or screws. Some studies reported that the retention type had no influence on peri-implant tissue[11, 61], whereas some studies reported that cement-retained prostheses have a greater area of microgap and higher bacterial loads than screw-retained prostheses[62].

### Conclusion

Our meta-analysis revealed that the implant restoration protocol using one-time abutment is superior to repeated abutment for platform-switched implant in terms of less bone resorption and soft tissue shifts. However, the clinical use must be prudent. Future randomised clinical trials should be conducted to compare the outcomes of one-time abutment and repeated abutment to further confirm these findings.

## Supporting information

S1 Table Consort checklists. (DOCX)

S2 Table PRISMA checklist. (DOC)

S3 Table Data availability. (DOCX)
