# Supplementary material for: One-time versus repeated abutment connection for platform-switched implant: A systematic review and meta-analysis
Source: PLoS One. 2017 Oct 19;12(10):e0186385. doi: 10.1371/journal.pone.0186385 (PMC5648164; doi:10.1371/journal.pone.0186385)
Supplement: S1 Table — (DOCX) [file pone.0186385.s001.docx]

|  | Grandi  et al.2012 | Grandi  et al.2014 | Degedi  et al.2011 | Degedi  et al.2014 | Canullo  etal.2010 | Koutouzis  etal. 2013 | Luongo  etal.2015 | Molina  etal.2016 |
| --- | --- | --- | --- | --- | --- | --- | --- | --- |
| **(Abstract: 1a)**  Identification as a randomised trial in the title | A  multicentre randomised controlled trials | A multicentre randomised controlled trial | **NM** | A randomized  controlled clinical study | A  randomized clinical trial | A short term randomized controlled clinical trial | A  multicentre randomised controlled trial | A prospective randomized clinical trial |
| **(Abstract: 1b):**  Structured summary of trial design, methods, results, and conclusions | **Page9** | **Page 141** | **Page**  **1303** | **Page1** | **Page285** | **Page807** | **Page129** | **Page1** |
| **(Introduction:**  **2a)**  Scientific background and explanation of rationale | **Page10** | **Page142** | **Page**  **1303 to1304** | **Page1 to 2** | **Page286** | **Page808** | **Page130** | **Page2** |
| **(Introduction:**  **2b)**  Specific objectives or hypotheses | **Page10:**  To compare crestal bone resorption around implants using definitive abutment versus provisional abutment later replaced by custom-made abutments. | **Page142:**  To assess if the placement of definitive abutments would reduce marginal bone loss. | **Page**  **1304:**  to see if non-removal of the abutment  placed at the time of the surgery would improve bone healing around the implants | **Page2:**  To assess if the nonremoval  of abutments  would improve bone and gingival healing . | **Page286:**  To evaluate  the influence on peri- implant marginal bone levels  using only definitive  abutments versus provisional abutments. | **Page808：**The aim of this study was to evaluate the impact of repeated healing abutment disconnection/reconnection on the soft and hard  Peri-implant tissues | **Page130:**  To compare hard and soft  tissue changes between definitive abutments  never removed  versus  provisional abutments changed at least three times | **Page2:**  To test the  hypothesis that placing the definitive abutment improve the stability of hard and soft  peri-implant tissues compared to placing a healing abutment and subsequent placement of the definitive abutment. |
| **(Methods:Trial design3a)**  Description of trial design (such as parallel, factorial) including allocation ratio | **Page11**:  A multicentre, randomised controlled trial…Twenty-eight patients were randomised to DA group and PA group (14patients each group) | **Page142 and 146**:  A multicentre,  randomised ,  parallel,  controlled trial… Half of patients receive the DA and half the PA | **Page1303 to 1304:**  A prospective study… Twelve patients were enrolled in control group. Twelve patients were enrolled in test group | **Page3:**  This study was designed as randomized controlled trial.  Control:35patients  Test:33  patients | **Page286:**  A  multicentre randomised  clinical trial of parallel group design | **Page808:**  This study is a prospective , randomized, controlled clinical trial | **Page131:**  The trial was designed as a multicentre randomised  controlled trial of parallel group design with two  arms. | **Page2:**  The study was designed as a prospective, randomized, controlled clinical trial with a parallel design. |
| **(Methods:Trial design3b)**  Important changes to methods after trial commencement (such as eligibility criteria), with reasons | **NM** | **NM** | **Page1306:**  Three cases  failed to achieve the minimal insertion torque  value and then treated with a one-stage approach. | **NM** | **NM** | **NM** | **NM** | **NM** |
| **(Methods:**  **Participants 4a**)  Eligibility criteria for participants | **Page11** | **Page 142** | **Page1304** | **Page2** |  | **Page808** | **Page131** | **Page2** |
| **(Methods:**  **Participants4b**)  Settings and locations where the data were collected | **Page11**:  In Italy | **Page 142 :**  In Italy | **Page1304:**  a private dental office in Bologna, Italy | **Page2:**  a private dental office in  Bologna, Italy | **Page286:**  private dental centresof Rome | **Page808:**  College of Dentistry, University of Florida | **Page131:**  Italian  private practices | **Page1:**  University Complutense of Madrid |
| **(Methods: Interventions5)**  The interventions for each group with sufficient details to allow replication, including how and when they were actually administered | **Page11 to 13** | **Page143 to146** | **Page1304 and 1305** | **Page 2 to 4** | **Page 286 to 291** | **Page 808 to 810** | **Page 131 to 135** | **Page 1 to 3** |
| **(Methods:**  **Outcomes6a)**  Completely defined pre-specified primary and secondary outcome measures, including how and when they were assessed | **Page12:**  (1)outcome measures  (6 and 12 months after surgery)  (2)  radiographic assesment  (6 and 12 months after surgery) | **Page144**:  *Primary outcome:* (1)crown/implant failure  *secondary outcome:*  (2)  complications  (3)marginal bone level changes | **Page1304:**  (1) Restoration success  (2) Implant success  (3) Bone healing  (4) Biological or technical complications and any  other adverse event | **Page3:**  (1)Biological or technical complications(2)soft tissue biotype of the patient  (3)Soft tissue dimensional changes  (4)changes of the bone | **Page 290:**  bone remodelling | **Page 808 to 809**  (1)clinical examinations  (2)radiographic exazamination | **Page133:**  **(1)** Prosthesis and implants failure  (2) Peri-implant marginal bone level changes  (3)Patient satisfaction | **Page 2 to 3**  **(1)** Clinical assessment  **(2)**Soft tissue margin  (3) Papilla fill  (4) Radiographic assessment |
| **(Methods:**  **Outcomes6b)**  Any changes to trial outcomes after the trial commenced, with reasons | **NM** | **NM** | **NM** | **NM** | **NM** | **NM** | **NM** | **NM** |
| **(Methods:Samplesize7a)**  How sample size was determined | **NM** | **Page145:**  No sample size calculation was performed. | **NM** | **NM** | **Page287:**  *priori* power analysis | **NM** | **NM** | **NM** |
| **(Methods:**  **Sample size7b)**  When applicable, explanation of any interim analyses and stopping guidelines | **NM** | **NM** | **NM** | **NM** | **NM** | **NM** | **NM** | **NM** |
| **(Methods:**  **Randomization8a)**Method used to generate the random allocation sequence | **Page11:**  Computerised random numbers | **Page146:**  Computer generated random  numbers. | **NM** | **Page3:**  Computerized  random  number generator | **Page287**:  random number generator  utility | **Page808:**  Computer-generated list | **Page135:**  computer generated restricted random lists | **Page2:**  The randomization sequence was created  using a computer-generated list |
| **(Methods:**  **Randomization8b)**  Type of randomisation; details of any restriction (such as blocking and block size) | **Page11:**  Simple randomization  procedures | **NM** | **NM** | **Page3:**  A nonrepeatable computerized  random  number generator | **Page287**:provided by astatistician unaware of  the study protocol using a random number generator  utility. | **Page 808:**  Computer-generated list | **Page135:**  computer generated restricted random lists | **Page2:**  The randomization sequence was created  using a computer-generated list using  Excel 2010 (Microsoft, Redmond, WA, USA)  with a 1 : 1 allocation using random block  sizes 4 by an independent research monitor |
| **(Methods: Allocation**9)  Mechanism used to implement the random allocation sequence (such as sequentially numbered containers), describing any steps taken to conceal the sequence until interventions were assigned | **Page11:**  A locked computer file | **Page146:**  A closed opaque envelope | **NM** | **Page3:**  A locked list | **Page287**:A closed envelope | **NM** | **Page132:** A sealed envelope | **Page2:** opaque-sealed envelopes |
| **(Methods:  Implementation 10)**Who independent research monitor sequence, who enrolled participants, and who assigned participants to interventions | **Page11** | **Page146** | **Page 1304 to 1305** | **Page3:**  computerized random  numbers.. | **Page 286 to 287:**  The randomi sation list was provided by a statistician | **Page 808:**  Computer-generated list...All the study procedures and data collection was by a periodontist. | Computer-generated list... | **Page 2:**  An independent research monitor independent research monitor.. Recruitment  was carried out by one of the researchers |
| **(Methods:**  **Blinding11a)**If done, who was blinded after assignment to interventions (for example, participants, care providers, those assessing outcomes) and how | **Page11 to 13:**  The surgeons and the experts of outcome measures were not blinded…  The persons of radiographic assessment were blinded. | **Page144**  **and 146**  The surgeons and assessors of primary  outcome  were not blind.  The assessors of secondary outcomes  were blinded. | **NM** | **Page3:**  The assessors of all outcomes were blind | **Page 287:**  The surgeon was not blind…  The assessor of outcome was blind | **Page809:**  (1) the assessor of clinical exzamination was not masked.  (2) the assessor of radiographic exzamination was masked. | **Page123:**  The surgeon was not blind…  The assessor of outcome was blind | **Page 2:**  The assessors of outcomes were blind |
| **(Methods:**  **Blinding11b)**  If relevant, description of the similarity of interventions | **Page 12**  **and 13** | **Page 144 and 145** | **NM** | **Page 2 and 3** | **Page 287 to 290** | **Page 808 to 810** | **Page131 to 135** | **Page1 to 4** |
| **(Methods: Statistical methods 12a)**  Statistical methods used to compare groups for primary and secondary outcomes | **Page13:**  Wilcoxon-Mann-Whitney test | **Page 146:**  *Primary outcomes:* chi-square test  *Secondary outcomes: t* test | **Page1305:**  *Bone level*:  Wilcoxon’s t-test  with a 95% confidence interval | **Page4:**  *Bone levels:* Mann-Whitney test with a 95% confidence interval  (*p* < .05) | **Page291:**The Student *t* test | **Page 810:**  The Fisher exact test.  Student t test. | **Page135:**  (1)Chi-square test or  Fisher’s exact test  (2) Mann-Whitney U and Kruskal-Wallis test | **Page4 :**  a two-way ANOVA and Shapiro–Wilk tests |
| **(Methods: Statistical methods 12b)**Methods for additional analyses, such as subgroup analyses and adjusted analyses | **NM** | **NM** | **NM** | **NM** | **NM** | **NM** | **NM** | **NM** |
| **(Results: Participant flow (13a)**  For each group, the numbers of participants who were randomly assigned, received intended treatment, and were analysed for the primary outcome | **Page14:**  *(1)Assessed for eligibility***:**34 patients  *(2)Randomization*: Control:14 patients  Test:14 patients  *(3)Analysed for the primary outcome:*  Control:14  Test:14 | **Page146:**  *(1)Assessed for eligibility :*  Control:14 Test:14  *(2)Randomization:*  Control:14 Test:14  *(3)Analysed for the primary*  *outcome:*  Control:13  Test:12 | **Page1306:**  A total of 48 implants thatfulfilled the inclusion  criteria…  Three cases were then dropped from the study | **Page4:**  *1)Assessed for eligibility :*91 patients  *(2)Randomization:*  Control:35 Test:33  *(3)Analysed for the primaryoutcome:*  Control:29  Test:24 | **Page292:**  *1)Assessed for eligibility :*32 patients  *(2)Randomization:*  Control:16  Test:16  *(3)Analysed for the primaryoutcome:*  Control:10  Test:15 | **Page811:**  Control:11 implants  Test: 10 implants | **Page135:**  *1)Assessed for eligibility :*80patients  *2)Randomization:*  Control:  40  Test:40  *(3)Analysed for the primaryoutcome:*  Control:  40  Test:40 | **Fig4:**  *1)Assessed for eligibility:41*  *2)Randomization:*  Control:21  Test:18  *(3)Analysed for the primaryoutcome*  Control:19  Test:16 |
| **(Results: Participant flow (a diagram is strongly recommended13b)**For each group, losses and exclusions after randomisation, together with reasons | **None** | **Page146:**  Three patients were excluded because of the poor postextractive  sockets | **Page1306:**  Three cases were then dropped because of the failure to achieve minimal insertion torque | **Page4 and5** | **Page292:**  Control:five cases( buccal bone fracture,  poor primary stability)  Test:one case(poor primary stability) | **Page 811:**  No losses and exclusions occured | NM | **Page4:**  One implant was lost  due to a premature failure in osseointegration. Two were excluded due  to lack of primary stability |
| **(Results:**  **Recruitment 14a)**Dates defining the periods of recruitment and follow-up | **Page14:**  *Periods of recruitment:* between Novenmber 2009 and March 2010  *Periods of follow-up:*baseline, 6 months, 12 months | **Page142:**  *Periods of recruitment*:  from October 2012 to February2013  *Periods of follow-up:*  (1)*Implant stability* :4  months after loading  *(2)Peri-implant marginal bone level changes:*  1 year after loading. | **Page1306:**  *Periods of recruitment*:between February  2006 and April 2007  *Periods of follow-up:*6 months, 1 year, 2 years, 3 years | **Page4 :**  *Periods of recruitment*:between July 2009 and  September 2010  *Periods of follow-up:*6 months, 1 year, 2 | **Page286:**  *Periods of recruitment*:September 2005 to December 2006  *Periods of follow-up:*  18months , 36 months | **Page808:**  *Periods of recruitment:* between 2010 and 2012.  *Periods of follow-up:*  3 months  6months | **Page136:**  *Periods of recruitment:* April 2010 to September 2012  *Periods of follow-up:*  4-months post-loading | *Periods of recruitment:*  from March 2011 to October 2012  *Periods of follow-up:*6months, 12months |
| **(Results:**  **Recruitment 14b)**  Why the trial ended or was stopped | **NM** | **NM** | **NM** | **NM** | **NM** | **NM** | **NM** | **NM** |
| **(Results:**  **Baseline data15)** A table showing baseline demographic and clinical characteristics for each group | **Table1:** Patients’ characteristics | **Table1:** Patients’ and interventions’ characteristics | **NM** | **NM** | **Table1:**  Characteristicsof patients included  in the test (DA) and  control(PA)groups. | **Table1:** Characteristicsof the patient sample | **Table1:** Patient and intervention characteristics. | **Table1:**  Baseline demographic characteristics of the study sample |
| **(Results:**  **Numbers analysed 16)**  For each group, number of participants (denominator) included in each analysis and whether the analysis was by original assigned groups | **Page 14:**  No drop-out occurred after randomization  (14:14) | **Page146:**  All nanlysis:  12:13  (Not original assigned groups. Three cases were excluded bofore follow-up) | **Page1306:**  All analysis:  24: 24 (Yes, original assigned groups. However,  three  cases were dropped because of failure to achieve minimal insertion torque  value ) | **Page5:**  All analysis:  Control:29  Test:24  (Not original assigned groups) | **Page 292 and 293 ：**  All analysis:  10:15 | **Page 810 and 811:**  All analysis:11:10 | **Page 136 and 138:**  All analysis:  40:40 | **Fig4**  All analysis:  19:16 |
| **(Results:**  **Outcomes and**  **estimation 17a)**For each primary and secondary outcome, results for each group, and theestimated effect size and its precision (such as 95% confidence interval) | **Page15:**  **Table3** | **Page147:**  **Table2 and Table3** | **Page 1306, Table 2 to 4** | **Page 6 to10**  **Table 1 to 6** | **Page 292 and 293 ：**  **Table 2 and Table 3** | **Page 810 and 811:**  **Table 3 to 5** | **Page 136 and 138**  **Table 2 and 3** | **Page 4 to7**  **Table 2 and 3** |
| **(Results:**  **Outcomes and estimation 17b)**  For binary outcomes, presentation of both absolute and relative effect sizes is recommended | **NM** | **Page146** | **Page1305** | **Page 4 to 6** | **NM** | **NM** | **Page137** | **NM** |
| **(Results:**  **Ancillary**  **analyses18)**  Results of any other analyses performed, including subgroup analyses and adjusted analyses, distinguishing pre-specified from exploratory | **NM** | **NM** | **NM** | **NM** | **NM** | **NM** | **NM** | **NM** |
| **(Results:Harms19)**  All important harms or unintended effects in each group | **None** | **Page146:**  peri-implant mucositis, abutment screw loosening | **Page1306:**  external  ecchymosis, substantial  discomfort | **Page6:**  moderate  sensory disturbances, gum irritation | **Page 292:**  without any serious complications or side effects | **NM** | **NM** | **Page4:**  lack of primary stability, premature failure in osseointegration |
| **(Discussion:**  **Limitations20)**Trial limitations, addressing sources of potential bias, imprecision, and, if relevant, multiplicity of analyses | **Page15:**  Only fucus on the effect on hard tissue | **Page148:**  smallsample size, unblinding | **Page 1307:**  Only fucus on the effect on har tissue | **Page9:**  unable to find a connection between the  amount of buccal recession and the biotype of thepatient | **Page295:**  the limited sample size and the inclusion of only  maxillary premolars | **Page813:** limited sample size, and short duration | **Page139:**  small sample size and the relatively short followup  duration. | **Page9:** implants in single and  multiple units were simultaneously combined  in the analysis. |
| **(Discussion:**  **Generalisability 21)**Generalisability (external validity,applicability) of the trial findings | **Page15** | **Page 147 and 148** | **Page1307** | **Page11** | **Page 294 and 295** | **Page813** | **Page139** | **Page 8 to 9** |
| **(Discussion:**  **Interpretation 22)**  Interpretation consistent with results, balancing benefits and harms, and considering other relevant evidence | **Page15** | **Page 147 and 148** | **Page1307** | **Page 10 and 11** | **Page 294 and 295** | **Page 812 and 813** | **Page139 to 140** | **Page 8 to 9** |
| Registration number and name of trial registry | NM | **NM** | **NM** | **NM** | **NM** | **NM** | **Page131:**  protocol number 187/09 |  |
| Where the full trial protocol can be accessed, if available | NM | **NM** | **NM** | **NM** | **NM** | **NM** | **Page131:**The study was approved on 17 December 2009 by  the ethical committee of the University of Naples,  Federico II(). |  |
| Sources of funding and other support (such as supply of drugs), role of funders | NM | **NM** | **Page 295:**  For invaluable technical support of Mr Gianluca Sighinolfi | **Page 11:**  For invaluable technical support of Mr Gianluca Sighinolfi | **Page 295:**  the skills and commitment of  Dr Audrenn Gautier and Dr Henry Canullo | **Page813** | **NM** | **Page9:**  partially funded by a  research project from the CAMLOG Foundation |
