# Supplementary material for: One-time versus repeated abutment connection for platform-switched implant: A systematic review and meta-analysis
Source: PLoS One. 2017 Oct 19;12(10):e0186385. doi: 10.1371/journal.pone.0186385 (PMC5648164; doi:10.1371/journal.pone.0186385)
Supplement: S2 Table — (DOC) [file pone.0186385.s002.doc]

| **Section/topic** | **#** | **Checklist item** | **Reported on page #** |
| --- | --- | --- | --- |
| **TITLE** | | |  |
| Title | 1 | One-Time versus Repeated Abutment Connection for Platform-Switched Implant: A Systematic Review and Meta-Analysis | Page1(Title page) |
| **ABSTRACT** | | |  |
| Structured summary | 2 | **Objectives:** The objective of this review was to compare peri-implant tissue changes(clinical and radiographic aspects) of the implant restoration protocol using one-time abutment to repeated abutment for platform-swithcing implant.  **Data souces**: Pubmed, Embase, Web of Science.  **Study eligibility criteria:** (1) RCTs and CCTs with a minimum of six monthes duration of follow-up. (2)Studies that compared peri-implant tissue changes in subjects with one-time abutment and subjects with repeated abutment. (3)Implant systems with the characters of internal connection and platform-switching. (4)Participants are≥18 years of age diagnosed with no chronic periodontitisand no history of systemic disease.  **Results:** One-time abutment group showed significantly better outcomes than repeated abutment group, as measured in the standardised differences in mean values (fixed- and random-effect model): vertical bone change(0.41, 3.23) in 6 months, (1.51, 14.81) in 12 months and (2.47, 2.47) in 3 years and soft tissue change (0.21,0.23). No significant difference was observed in terms of probing depth and complications.  **Conclusions and implications of key findings:** Our meta-analysis revealed that implant restoration protocol using one-time abutment is superior to repeated abutment for platform switched implant because of less bone resorption and soft tissue shifts in former. However, future randomised clinical trials should be conducted to further confirm these findings because of the small samples and the limited quality of the original research.  **Limitations**: limited number of eligible studies; difference was very subtle between the two groups in clinic view. | Page2(Abstract) |
| **INTRODUCTION** | | |  |
| Rationale | 3 | Dis/re-connected abutment manipulation may disturb the implant–mucosal barrier, that is, disturbance of the zone of ‘junctional epithelium and connective tissue integration’, and further affect the marginal peri-implant tissues, including the peri-implant bone, and finally affect the stability of the peri-implant tissue^12^. On the contrary, another restorative protocol is ‘one-time abutment’, which means definitive abutment is connected to the implant once implant is exposed into the oral environment. The definitive abutment is retained during all the procedures of the final prosthesis fabrication and no healing abutment is needed. | Page4(Introduction) |
| Objectives | 4 | The present review aims to statistically analyze the clinical outcomes of one-time abutment compared to repeated abutment for platform-switching implant system, and provided higher level of evidence for implant therapy.  **PICO format:**  **Population or participants:** Patients that needimplant restorations **Intervention:** one-time abutment during implant restorations **Comparison:** repeated abutment during implant restorations  **Outcome:** peri-implant tissue changes (clinical and radiographic aspects) | Page 6 (introduction and Materials and methods  ) |
| **METHODS** | | |  |
| Protocol and registration | 5 | Indicate if a review protocol exists, if and where it can be accessed (e.g., Web address), and, if available, provide registration information including registration number. | None |
| Eligibility criteria | 6 | **Inclusion criteria:**  1, Randomized controlled trials (RCTs) and clinical controlled trials (CCTs) with a minimum of six monthes duration of follow-up after abutment connection to implant body.  2, Studies that compared peri-implant tissue changes in subjects with one-time abutment and subjects with repeated abutment.  3, Implant systems with the characters of internal connection and platform-switching.  4, Participants are≥18 years of age diagnosed with no chronic periodontitis and no history of systemic disease. | Page7(Materials and methods) |
| Information sources | 7 | Web of Science, Pubmed, and Embase(updated until August 15, 2017) | Page7(Search strategies) |
| Search | 8 | Pubmed:  1, ((“provisional” AND Dental Abutment [Mesh]) OR (“temporary” AND Dental Abutment [Mesh]) OR (“healing” AND Dental Abutment [Mesh]) OR (“repeated” AND Dental Abutment [Mesh]) OR (“disconnection” AND “reconnection” AND Dental Abutment [Mesh]) OR (“removal” AND Dental Abutment [Mesh]))  2, ((“non-removal” AND Dental Abutment [Mesh]) OR (“final” AND Dental Abutment [Mesh]) OR (“definitive” AND Dental Abutment [Mesh]) OR (“standard” AND Dental Abutment [Mesh]) OR (“immediate” AND Dental Abutment [Mesh]) OR (“one-time” AND Dental Abutment [Mesh]) OR (“one time” AND Dental Abutment [Mesh]))  3, #1 AND #2 | Page 7 and 8(Search strategies) |
| Study selection | 9 | **Selection of study**: The titles and abstracts of all articles acquired from the electronic search were screened independently by two authors. Irrelevant studies were discarded. The full text of potentially relevant articles obtained from the above search strategies were screened by two reviewers. Papers were excluded if they were case report, conference proceedings, reviews, animal studies and *in vitro* studies. Discrepancies were resolved by discussion between the reviewers. Only RCTs and CCTs that compared one-time abutment with repeated abutment and reported data on peri-implant tissue changes with a follow-up period of at least six months were selected and formed the base of this systematic review. | Page8(Materials and methods) |
| Data collection process | 10 | The search generated a total of 1640 papers. After screening the titles and abstracts in the first round, 29 papers were identified as relevant. After retrieving the full text, 21 of them were further excluded. Five of them were animal studies[^7,35-38^], two were in *vitro* studies[^39,40^], one is a review[^33^] and 11 were case reports and case series[^32,41-50^]. One study [^29^] reported implants with external connection and nonplatform switching. One study [^30^] did not provide relevant data that we need. In this study, authors compared a friction fit abutment (test group) with a conventional healing abutment (control group), and in both groups, abutments were dis-/reconnected several times. A total of eight papers[^21-28^] were finally included in this systematic review. | Page11(Materials and methods) |
| Data items | 11 | From the studies included in the final analysis, the following data were extracted (when available): year of publication, study design, implant surface, single- or multicentre study, number of implants, patient age, follow-up, antibiotic prophylaxis, use of mouthrinse, time from implant surgery to final restorations, implants sites, the relationship between the implant platform and the crest bone, stage of implant surgery, implant system, implant design, abutment manipulation, type of final prosthodontic retention, primary and secondary outcome, and main conclusions.  Primary outcomes:  *Vertical peri-implant bone changes*: Difference in vertical distance between implant platform level and the most coronal bone contacting with implant surface (mm)  *Horizontal peri-implant bone changes:* Difference in horizontal distance between the implant surface and the inner wall of the socket at implant platform level (mm). This measurement had positive or negative values depending on the presence of a gap (negative) or implant platform bone overgrowth (positive).  *Peri-implant soft tissue changes*:Difference in vertical distance between peri-implant highest buccal mucosa margin in maxillary or lowest buccal mucosa margin in mandible to the most coronal part of the prosthesis (healing abutment, the provisional restorations or the definitive restorations)(mm).  Secondary outcomes:  *Probing depth:* Difference in probing depth (mm)  *Postsurgical complications*: Difference in occurrence of pain, swelling, mucositis after surgery, or sensory disturbance | Page8 and 9 (Materials and methods) |
| Risk of bias in individual studies | 12 | The qualities of the included RCTs and CCTs were assessed in accordance with the recommendations of Cochrane Collaboration. The following terms were used to determine biases in the included studies: 1) selection bias refers to sequence generation and allocation concealment; 2) performance and detection bias refer to blinding of participants and outcome assessors; 3) attrition bias refers to incomplete outcome data; 4) reporting bias refers to selective report of outcome. | Page10 (Materials and methods) |
| Summary measures | 13 | Dichotomous data were expressed as risk ratios (RRs) with 95% confidence interval (CI). Continuous data were expressed as standardised mean difference (SMD) with 95% CI. | Page10(Materials and methods) |
| Synthesis of results | 14 | Forest plots for each meta-analysis presented the original data of outcomes (displayed as blocks) with their respective CIs (displayed as lines), heterogeneity statistic (I^2^) and the pooled data of outcome (as rhomboid). Summary effects were calculated via both random- and fixed-effect models using Review Manager 5.3. The time points of bone changes and complications were from implant surgery, whereas the peri-implant soft tissue shifts and probing depth changes were from loading after mucosal detumescence. The plus sign represents bone growth or coronal soft tissue shifts, whereas the minus sign indicates bone resorption or apical soft tissue shifts. The outcomes of horizontal and vertical bone changes were divided into subgroups according to their respective follow-up periods. Meta-analyses were performed when the included studies reported the same outcome measures with the similar follow-up periods | Page10(Materials and methods) |
|  |  |  |  |

Page 1 of 2

| **Section/topic** | **#** | **Checklist item** | **Reported on page #** |
| --- | --- | --- | --- |
| Risk of bias across studies | 15 | Specify any assessment of risk of bias that may affect the cumulative evidence (e.g., publication bias, selective reporting within studies). | Non |
| Additional analyses | 16 | The outcome of vertical bone changes were divided into subgroups according to their respective follow-up periods. Meta-analyses were performed when the included studies reported the same outcome measures with the similar follow-up periods. | Page13(Materials and methods) |
| **RESULTS** | | |  |
| Study selection | 17 | 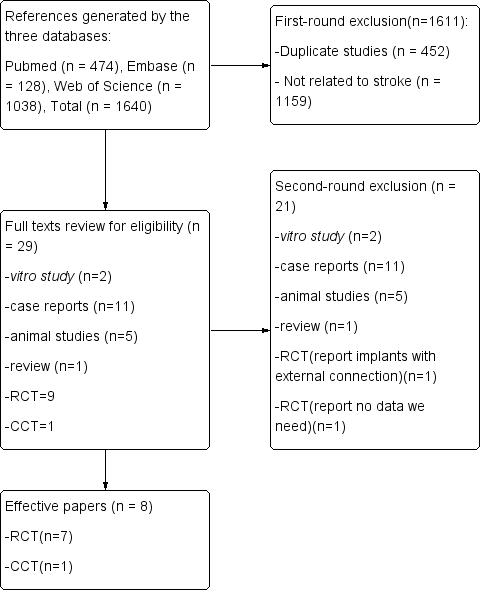 | Fig1 |
| Study characteristics | 18 | Study design and patient features:  A total of 197 implants were placed in the one-time abutment group, whereas 214 implants were included in the repeated abutment group. Among eight eligible studies, seven studies were RCTs[^21,22,24-28^]and one study[^23^] was CCT. Four studies [^21,24,25,27^] were multicentre RCTs, whereas the rest [^22,23,26,28^] were unspecified its study sites. The minimum duration of follow-up period was 6-months. All of the eligible studies reported patient age, and most of the participants were middle-aged persons. All participants in the eligible studies were systemic healthy subjects without diabetes, osteoporosis and other systemic disease which may influence the quality of implantation. Six studies reported that participants smoked less than 10 cigarettes, one study less than 20 cigarettes, one study did not mentioned smoking.  Installation site and restoration characteristics:  The implant systems included *Straumann Bone Level (Straumann*, *Switzerland)*; *Camlog*, *Conelog Screw-Line (Basel*, *Switzerland)*; *JDEvolution*, *JdentalCare (Modena*, *Italy)*; *Global Implants (Sweden & Martina*, *Padua*, *Italy)* and *Ankylos (Friadent*, *Germany*). All implants are with an internal implant–abutment connection and platform switching. Information regarding the length, diameter and shape of the implants were also provided. One study[^22^] evaluated implants inserted in the anterior maxillary regions, five evaluated implants placed in posterior regions[^21,23,25,26,28^], and two did not mentioned[^24,27^]. Four studies reported implantation in healed sites[^23,24,26,28^], whereas three studies reported immediate implantation[^21,22,25^], and one study reported implants placed in immediate extraction sockets or healed sites[^27^]. All of the implants underwent two to six months of healing before final restorations. Four studies reported that implants were placed beneath the bone crest [^22,23,25,27^] and four at bone level [^21,24,26,28^]. Three studies reported screw-retained restorations [^23,25,28^], two reported cemented [^24,26^], three did not mention [^21,22,27^]. In all studies, abutments were disconnected and reconnected thrice, including impression making, the metal framework and biscuit fitting and the delivery of the definitive prosthesis. | Page10 and11(results) |
| Risk of bias within studies | 19 | Each trial was assessed for risks of bias, and the results are summarised in Table 2 and Fig2. Among the eight studies that met the inclusion criteria, four studies clearly described the random sequence generation[^21,24,26,28^], and two studies described the allocation concealment clearly[^24,28^]. Seven studies reported that participants were blinded [^21-23,25-28^]. Six studies reported that outcome assessors were blinded [^21-23,26-28^]. For the incomplete outcome, five studies described some exclusions of participants, in which they need not to be considered as leading to missing outcome data [^22,24-27^]. Three studies [^22,26,28^] reported peri-implant soft and hard tissue changes. The drop-out rates were less than 45% in all studies. The common reasons for attrition were failure to achieve oral hygiene, lack of initial insertion torque and unsuitable extraction sockets. All of these conditions can result in bias. | Page15(results) |
| Results of individual studies | 20 | 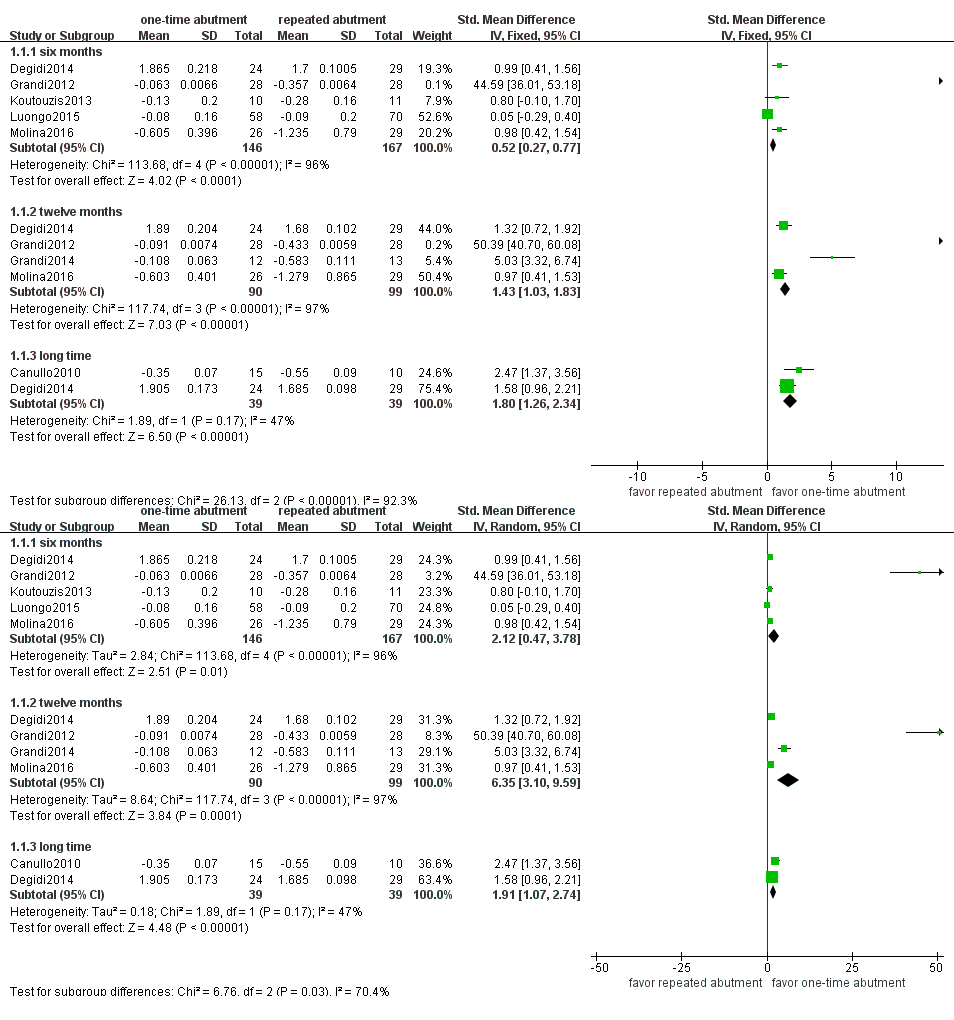  Figure 3. Forest plot of vertical bone changes  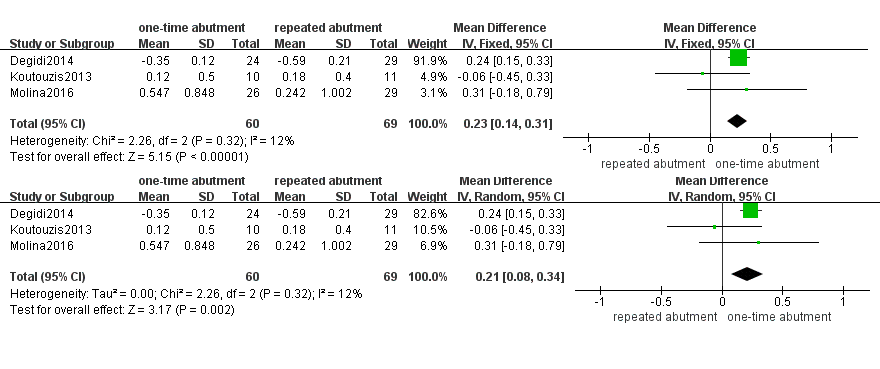  Figure 4. Forest plots of peri-implant soft tissue shifts  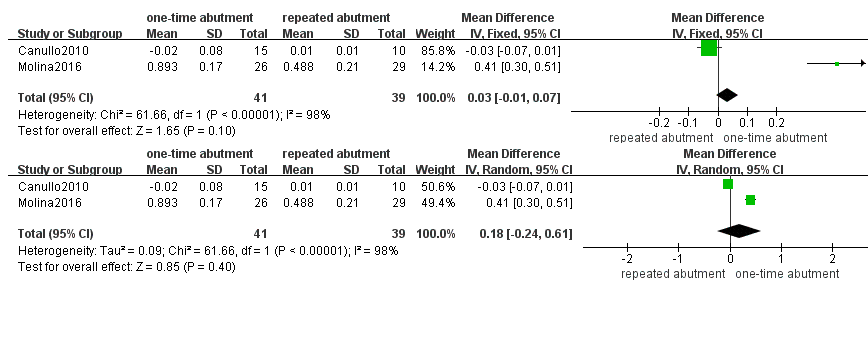  Figure 6. Forest plots of probing depth  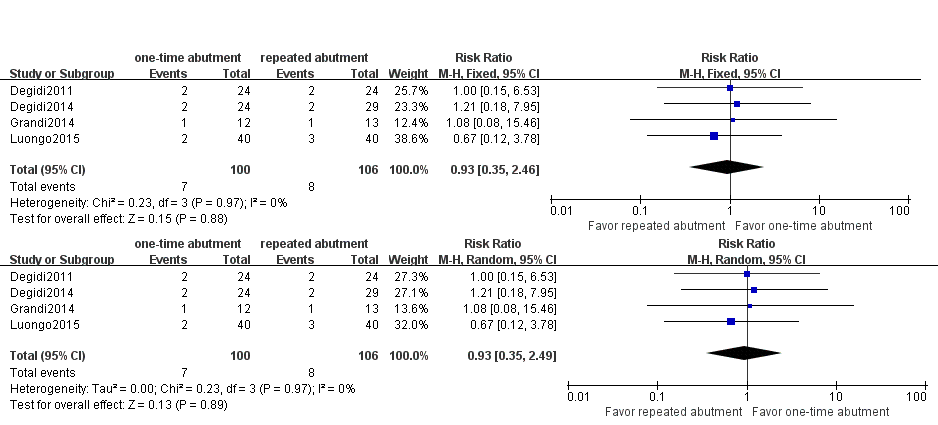  Figure6. Forest plots of post-surgical complications | Fig3-7  Fig3-6 |
| Synthesis of results | 21 | **Vertical bone changes:**  ***6-month subgroup***:the SMD (95% CI) of vertical bone resorption was 0.41(0.12, 0.69) in fixed model(p<0.01), and 2.12 3.23 (0.91, 5.56) in random model(p<0.01).  ***12-month subgroup***: the SMD(95% CI)of vertical bone resorption was 1.51(0.98, 2.05) in fixed model (p<0.00001) and 14.81 (6.52, 23.11) in random model (p<0.001).  ***3-year subgroup:*** the SMD (95% CI) of vertical bone resorption was 2.47 (1.37, 3.56) in fixed model (p<0.00001) and 2.47(1.37, 3.56) in random model (p<0.00001).  The results of subgroup analysis indicated that one-time abutment can significantly reduce vertical bone resorption compared to repeated abutment.  **Peri-implant soft tissue shift**: The SMD of peri-implant soft tissue shift was 0.23(0.14,0.31) in fixed model(p< 0.00001), and 0.21(0.08,0.34) in random model (p<0.01). The analysis revealed that one-time abutment significantly increase coronalperi-implant soft tissue shift compared to repeated abutment.  **Probing depth**: the SMD of increase in probing depth was 0.03(-0.01,0.07) in fixed model(p>0.05), and 0.18(-0.24,0.61) in random model(p>0.05).  **Post-surgical complications:**The mete-analysis did not find any significant difference between one-time and repeated abutment for risk of post-surgical complications [RR: 0.93(0.35,2.46), p>0.05 in fixed model; and 0.93(0.35,2.49), p>0.05 in random model]. | Page20 and 21(results) |
| Risk of bias across studies | 22 | Among the eight studies that met the inclusion criteria, four studies clearly described the random sequence generation[^21,24,26,28^], and two studies described the allocation concealment clearly[^24,28^]. Seven studies reported that participants were blinded [^21-23,25-28^]. Six studies reported that outcome assessors were blinded [^21-23,26-28^]. For the incomplete outcome, five studies described some exclusions of participants, in which they need not to be considered as leading to missing outcome data [^22,24-27^]. Three studies [^22,26,28^] reported peri-implant soft and hard tissue changes. The drop-out rates were less than 45% in all studies. The common reasons for attrition were failure to achieve oral hygiene, lack of initial insertion torque and unsuitable extraction sockets. All of these conditions can result in bias. | Page 18 (Results) |
| Additional analysis | 23 | 1,Studies on vertical peri-implant bone changes were divided into three subgroups, namely, 6month, 12month and long-term period(>12months)  2,The studies on horizontal bone change were divided into 6-month, 12-month, and long-time (>12 months) subgroups | Page20 and 21 (Results) |
| **DISCUSSION** | | |  |
| Summary of evidence | 24 | 1, Our meta-analysis revealed that one-time abutment resulted in significantly less vertical bone and soft tissue changes.  2, For probing depth, no significant difference between one-time and repeated abutment was observed.  3, For postsurgical complications, no significant difference between one-time and repeated abutment was observed. | Page22 to 24(disscussion) |
| Limitations | 25 | 1, Significant heterogeneity was detected among individual studies in meta-analyses on peri-implant vertical bone resorption and soft tissue shifts. The presence of statistical heterogeneity may be due to the low power of statistical test because only few studies were included in the aforementioned meta-analyses.  2, Given the limited number of eligible studies, meta-regression analyses were not conducted to address some important confounding factors, including implant level, implant sites and prosthodontics retention type, associated with outcomes.  3, Finally, the explanation of statistic significant difference in clinical use must be prudent because the difference was very subtle between the two groups in a clinical perspective. | Page23 and 24(disscussion) |
| Conclusions | 26 | Our meta-analysis revealed that the implant restoration protocol using one-time abutment is superior to repeated abutment for platform-switched implant in terms of less bone resorption and soft tissue shifts. However, the clinical use must be prudent. Future randomised clinical trials should be conducted to compare the outcomes of one-time abutment and repeated abutment to further confirm these findings. | Page19 8d00000000000000000000000000000000000000000000000000000000000000000000000000000000000000000000000000000000000000000000000000000(conclusion) |
| **FUNDING** | | |  |
| Funding | 27 | This paper has been prepared without any sources of institution, privateor corporate financial support,and there are no potential conflictsof interest. | Acknowledgements |

*From:*  Moher D, Liberati A, Tetzlaff J, Altman DG, The PRISMA Group (2009). Preferred Reporting Items for Systematic Reviews and Meta-Analyses: The PRISMA Statement. PLoS Med 6(7): e1000097. doi:10.1371/journal.pmed1000097

For more information, visit: **www.prisma-statement.org**.

Page 2 of 2
