# Supplementary material for: One-time versus repeated abutment connection for platform-switched implant: A systematic review and meta-analysis
Source: PLoS One. 2017 Oct 19;12(10):e0186385. doi: 10.1371/journal.pone.0186385 (PMC5648164; doi:10.1371/journal.pone.0186385)
Supplement: S3 Table — (DOCX) [file pone.0186385.s003.docx]

**Table1. Original data of peri-implant bone change**

|  | 6months | 12 months | 3 years |  |  |  |
| --- | --- | --- | --- | --- | --- | --- |
| Grandi2012 | DA:-0.063(0.0066)  PA:-0.357(0.0064) | DA:-0.091(0.0074)  PA:-0.433(0.0059) | NM |  |  |  |
| Koutouzis3013 | DA:-0.13(0.2)  PA:-0.28(0.16) | NM | NM |  |  |  |
| Luongo2015 | DA:-0.08(0.16)  PA:-0.09(0.2) | NM | NM |  |  |  |
| Molina 2016 | DA:-0.605(0.396)  PA:-1.235(0.79) | DA:-0.603(0.401)  PA:-1.279(0.865) | NM |  |  |  |
| Grandi2014 | NM | DA:-0.108(0.063)  PA:-0.583(0.111) | NM |  |  |  |
| Canullo2010 | NM | NM | DA:  -0.35(0.07)  PA:-0.55(0.) |  |  |  |
| **NM: not mentioned**  **DA: definitive abutment group PA:provisional abutment group**  **Mean(SD)(mm)** |  |  |  |  |  |  |
|  | | | |  |  |  |
|  |  |  |  |  |  |  |

**Table2. Original data of peri-implant soft tissue change**

|  | One-time abutment | Repeated abutment |
| --- | --- | --- |
| Degedi2014 | -0.35(0.12) | -0.59(0.21) |
| Koutouzis3013 | 0.12(0.5) | 0.18(0.4) |
| Molina2016 | 0.547(0.848) | 0.242(1.002) |
| **Mean(SD)(mm)** |  |  |

**Table3. Original data of probing depth**

|  | One-time abutment | Repeated abutment |
| --- | --- | --- |
| Canullo2010 | -0.02(0.08) | 0.01(0.01) |
| Molina2016 | 0.893(0.17) | 0.488(0.21) |
| **Mean(SD)(mm)** |  |  |

**Table4. Original data of postsurgical complications**

|  | One-time abutment | Repeated abutment |
| --- | --- | --- |
| Degedi2011 | 2/24 | 2/24 |
| Degedi2014 | 2/24 | 2/29 |
| Grandi2014 | 1/12 | 1/13 |
| Luongo2015 | 2/40 | 3/40 |
| **Events/Total** |  |  |
